# Complete mitochondrial genome sequencing of central Indian domestic pig

**DOI:** 10.1080/23802359.2016.1197077

**Published:** 2017-01-04

**Authors:** Ajit Pratap Singh, Kajal Kumar Jadav, Dharmendra Kumar, Nidhi Rajput, Awadh Bihari Srivastav, Bikash Chandra Sarkhel

**Affiliations:** a Animal Biotechnology Centre, Nanaji Deshmukh Veterinary Science Univeristy, Jabalpur, India;; b Centre for Wildlife Forensic and Health, Nanaji Deshmukh Veterinary Science Univeristy, Jabalpur, India

**Keywords:** *Sus scrofa domesticus*, mitogenome, next-generation sequencing, PGM Ion torrent

## Abstract

Domestic pig (*Sus scrofa domesticus*) is one of the important farm animal contributing 7% of the country’s total animal protein sources in India. In the present study, random hexamer primer was used to amplify the complete mitochondrial genome of Central Indian domestic pig and resolved the complete mitochondrial sequence by shotgun sequencing followed by *de novo* assembly in MIRA version 4.0.5. The sequence assembly revealed to be 15,827 bp mitogenome of pig (accession no. KT965278). The mitogenome in the present study has 99% homology with previously reported mitogenome of pigs from different parts of the world. The present study is the first report of complete sequence of mitogenome of pig from Indian subcontinent. Mitogenome analysis by MITOS web server revealed similarity of gene order, organization with the other pig breeds and vertebrates, comprising of 13 protein-coding genes, 22 tRNAs, 2 rRNAs and a control region. It was concluded that modified random hexamer can be successfully used for whole mitogenome sequencing using NGS without designing mitogenome-specific primer, thereby reducing cost and labor.

Pig (*Sus scrofa domesticus*) is domesticated since 8000 years, yet the origin and evolution of domestic pigs of India has not been established. The introgression of exotic genome under state sponsored programs to upgrade the local population using semen or live animals of exotic breeds has diluted the gene pool of local breeds, thus breed specification of the local domestic pig remains questionable. Most studies on Indian domestic pig mitochondrial DNA (MtDNA) are restricted to capillary electrophoresis (CE)-derived short sequence fragments analysis of cytocgrome b, 12S rRNA (Jadav et al. 2014a,b), D-loop region of mitogenome (Srivastava et al. 2014). There are no reports on characterization of the complete mitogenome of any of the domestic pigs of India. In this study, we have resolved the complete mitogenome of a domestic pig from Central India using PGM Ion torrent next-generation sequencing (NGS) technology. The ancestral history of Central Indian (CI) domestic pig was derived from GenBank, which retrieved 59 complete mitogenome sequences of pig and *Phacochoerus africanus* (NC 008830) as out group using MEGA 6 (Tamura et al. 2013). DNA extract was amplified using phosphorothioate-modified random hexamers (Thermo Fisher scientific) in a thermocycler and PCR product corresponding to the size of mtDNA gel purified using gel extraction kit (QIAGEN, Germany). Shotgun libraries were prepared from amplified mitogenome of one CI domestic pig and subjected to sequencing using NGS on PGM Ion torrent platform as per manufacturer’s instructions. A total of 90.1 MB raw data were obtained by shotgun sequencing corresponding to estimated 61.69× mitogenome coverage. A total of 184,527 reads were obtained and subjected to *de novo* assembly in Ion suite with plugin MIRA version 4.0.5 to obtain 41 contigs with N50 value of 2360, the largest contig observed was 16,827 bp (accession no. KT965278). The 16,827 bp length of the mitogenome of the CI domestic pig showed 99% homology with the available mitogenome sequences in the GenBank and genome annotation was performed using MITOS web server (Bernt et al. 2013). Evolutionary history of the CI domestic pig was inferred using the neighbor-joining (NJ) method (Saitou & Nei 1987) and the derived phylogenetic tree could clearly distinguish the pig sequences in two clades (Asian and European) by geographical definition. The CI domestic pig is within the Asian clade with distinct genetic makeup from Chinese subclade (Figure 1). Majority of the genes were encoded on the H-strand except ND6, COX3-1 gene and two tRNA genes (tRNAPro and tRNAGlu) were encoded on the L-strand. The length of tRNA genes varied from 65 to 75 bp with a control region of 1064 bp long. There were 9 overlaps and 20 intergenic spacer regions. The intergenic spacer region between tRNACys and tRNAAsn was of 31 bp length. The percent base composition of the heavy strand of the domestic pig of Central India was: A: 25.71, C: 13.31, G: 26.26, T: 34.72 and A + T content: 60.43. In all the protein-coding genes, ATG was the start codon except ND3, ND4, ND5, NAD1 and NAD2, where ATA was the start codon.

**Figure 1.**
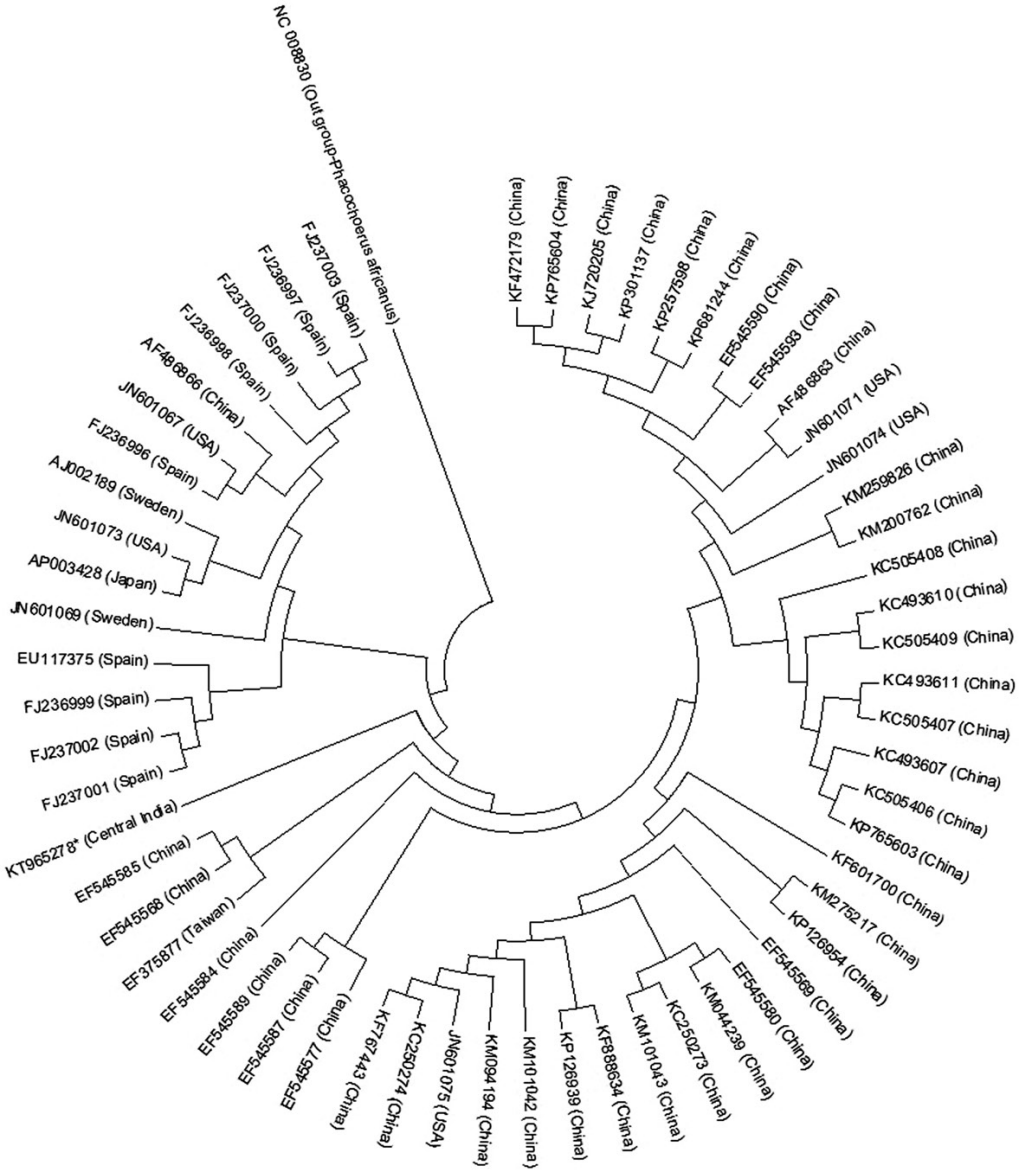
NJ-tree of complete mitochondrial sequences of 59 pigs and *Phacochoerus africanus* as out group (*present study from NGS).
